# Mapping the immune microenvironment for mandibular alveolar bone homeostasis at single-cell resolution

**DOI:** 10.1038/s41413-021-00141-5

**Published:** 2021-03-15

**Authors:** Weimin Lin, Qiwen Li, Danting Zhang, Xiaohan Zhang, Xingying Qi, Qian Wang, Yaqian Chen, Caojie Liu, Hanwen Li, Shiwen Zhang, Yuan Wang, Bin Shao, Li Zhang, Quan Yuan

**Affiliations:** 1 State Key Laboratory of Oral Diseases & National Clinical Research Center for Oral Diseases, West China Hospital of Stomatology, Sichuan University, Chengdu, China; 2 Department of Oral Implantology, West China Hospital of Stomatology, Sichuan University, Chengdu, China; 3 Department of Respiratory and Critical Care Medicine, Frontiers Science Center for Disease-related Molecular Network, Center of Precision Medicine, Precision Medicine Key Laboratory of Sichuan Province, West China Hospital, Sichuan University, Chengdu, China

**Keywords:** Bone, Bone quality and biomechanics

## Abstract

Alveolar bone is the thickened ridge of jaw bone that supports teeth. It is subject to constant occlusal force and pathogens invasion, and is therefore under active bone remodeling and immunomodulation. Alveolar bone holds a distinct niche from long bone considering their different developmental origin and postnatal remodeling pattern. However, a systematic explanation of alveolar bone at single-cell level is still lacking. Here, we construct a single-cell atlas of mouse mandibular alveolar bone through single-cell RNA sequencing (scRNA-seq). A more active immune microenvironment is identified in alveolar bone, with a higher proportion of mature immune cells than in long bone. Among all immune cell populations, the monocyte/macrophage subpopulation most actively interacts with mesenchymal stem cells (MSCs) subpopulation. Alveolar bone monocytes/macrophages express a higher level of Oncostatin M (Osm) compared to long bone, which promotes osteogenic differentiation and inhibits adipogenic differentiation of MSCs. In summary, our study reveals a unique immune microenvironment of alveolar bone, which may provide a more precise immune-modulatory target for therapeutic treatment of oral diseases.

## Introduction

Due to tooth-derived inflammatory response and occlusal stress stimuli, the metabolism and remodeling of alveolar bone are considered to be the most active among the entire skeletal system.^1–4^ Alveolar bones have a different developmental origin and ossification process compared with long bones and other bones.^5^ Craniofacial bone marrow mesenchymal stem cells (MSCs) show higher proliferation rate and osteogenic differentiation potential, but lower chondrogenic and adipogenic differentiation capability.^6,7^ Interestingly, alveolar bone is more resistant to bone loss and adipocytes accumulation than long bone in ovariectomized rodents.^8,9^ Alveolar bone contacts with the external microenvironment through the periodontal barrier, which exerts important regulatory effects on immune homeostasis of alveolar bone.^10^ Although accumulating evidence demonstrated unique physiological characteristics of alveolar bone, there is a lack of systematic description of alveolar bone cell heterogeneity and the difference from long bone.

Single-cell RNA sequencing (scRNA-seq) makes it be possible to analyze tissue heterogeneity at the level of individual cell and explore the contribution of different cell subtypes to physiological function and pathogenesis. Recently, two independent studies constructed complete single-cell atlas of long bone stromal cells with fluorescence-activated cell sorting (FACS).^11,12^ Baccin et al. further explored the spatial heterogeneity of bone marrow stromal cells with laser capture microdissection technique.^13^

Besides stromal cells, the skeletal system is also the largest reservoir of haematopoietic lineages, which contains haematopoietic stem cells, lymphoid/myeloid progenitors, and mature immune cells. These immune cells interact with bone marrow stromal cells, thereby regulating the homeostasis of skeletal system.^14^ The regulatory effect of macrophages on bone stromal cells has been confirmed.^15,16^ Mice depleted of macrophages showed an osteoporotic phenotype.^17^ In aged mice that received bone marrow macrophages transplantation from young mice, transplanted macrophage infiltration was observed at the fracture site and the healing of fracture was accelerated.^18^ Although previous scRNA-seq studies have constructed single-cell atlas of long bone marrow cells, study focusing on the regulation of immune cell populations on bone marrow stromal cells and bone homeostasis through scRNA-seq is still lacking.

In this study, we perform scRNA-seq on mouse mandibular alveolar bone and reveal that macrophages are the largest cell population that interacts with MSCs. Compared with long bone marrow (LBM), the proportion of activated macrophage subcluster in alveolar bone marrow (ABM) is higher, and this subcluster is the major group that secretes cytokine Oncostatin M (Osm). ABM macrophage-conditioned medium more effectively promotes osteogenic differentiation and inhibits adipogenic differentiation of MSCs through an Osm-dependent pathway.

## Results

### Characterization of mandibular alveolar bone single-cell atlas

**Fig. 1:**
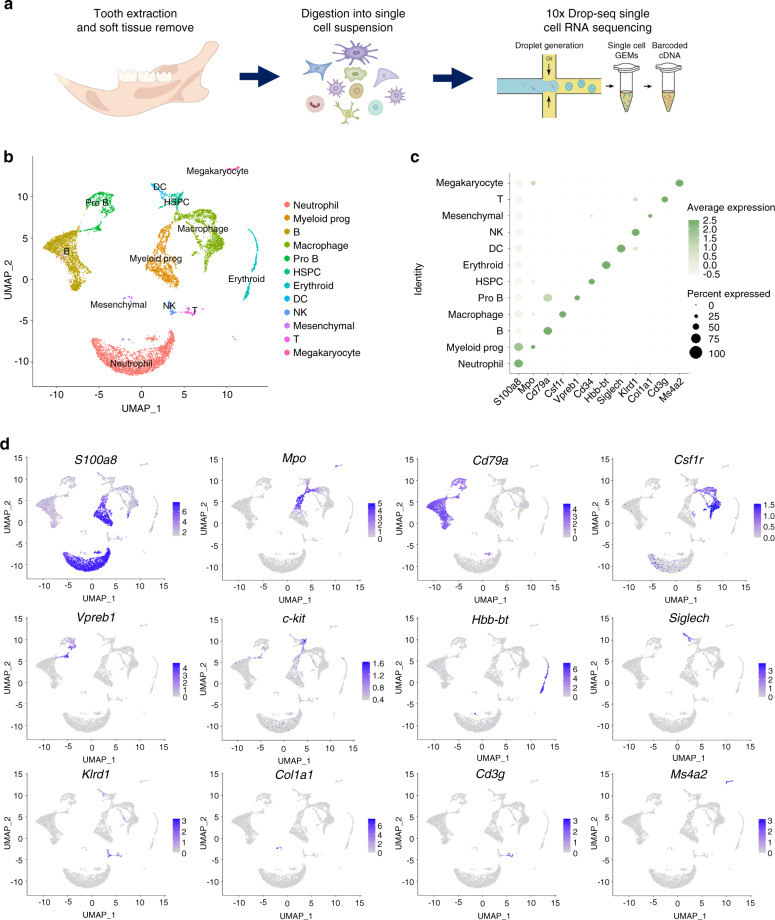
Characterization of the single-cell atlas of alveolar bone. **a** Flow chart of preparation of scRNA-seq samples from mouse mandibular alveolar bone. **b** Cells identified by scRNA-seq were visualized with UMAP. Different cell populations were defined and distinguished by color. Each point represented an independent cell. **c** Specific expression of marker genes in different cell types. **d** The expression levels of marker genes were projected onto UMAP atlas

The mouse mandibular alveolar bones were dissected for enzymatic digestion and subjected to droplet-based scRNA-seq (Fig. 1a). A total number of 10 224 cells were obtained with 29 053 genes of each cell. We preprocessed the dataset with Seurat package. The median value of feature_RNA is between 1 000 and 2 000, and cells with mitochondrial gene expression higher than 25% are filtered out (Fig. S1a, b). After quality control, 2 000 genes with the most variable value from 29 053 genes were selected for subsequent analysis (Fig. S1c). Then the UMAP method was applied to reduce the dimensionality (Fig. S1c). Cells were divided into 12 subgroups based on classic cell surface markers (neutrophil—*S100a8*, myeloid progenitor—*Mpo*, macrophage—*Csf1r*, dendritic cell—*Siglech*, B cell—*Cd79a*, pro-B cell—*Vpreb1*, T cell—*Cd3g*, natural killer cell—*Klrd1*, megakaryocyte—*Ms4a2*, erythrocyte—*Hbb-bt*, hematopoietic stem and progenitor cell—*Cd34*, and mesenchymal cell—*Col1a1*) (Fig. 1c, d). The top 5 expressed genes expressed in each defined cell type were identified and compared (Fig. S1d).

Since non-immune cells (mesenchymal stromal cells) accounted for only 1.74% of all identified cells, these cells were divided into a small cluster for identification. They are divided into four subclusters (Fig. S2a), categorized as MSCs (*Lepr*^+^), osteoblasts (*Bglap*^+^), endothelial cells (*Cdh5*^+^), and neurological cells (*Plp1*^+^) (Fig. S2b, c). As expected, chondrocyte was not identified in alveolar bone stromal cells, which is different from that of long bone.

### Identification of cell–cell interaction in alveolar bone microenvironment

**Fig. 2:**
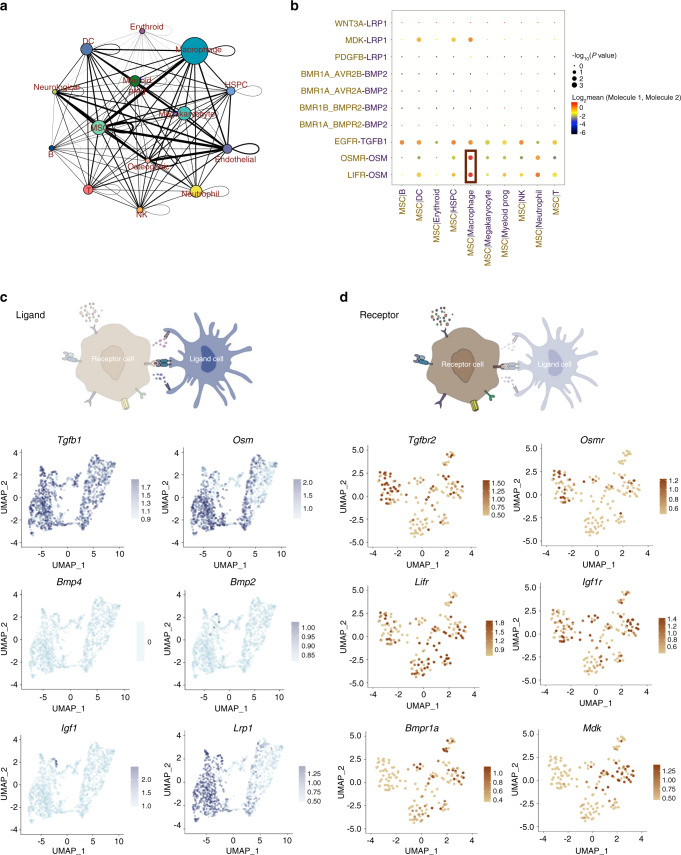
Cell–cell interaction between immune cells and stromal cells in alveolar bone marrow. **a** Network diagram of the cell–cell interaction of different cells in the alveolar bone marrow. The size of the circle represented the number of interactions with all other types of cells, and the thickness of the line represented the interaction number of cells between the line. **b** Visualization of the selected macrophage-MSC crosstalk pathway. **c** Expression of the ligands in monocytes/macrophages. **d** Expression of the receptors in stromal cells (MSC, osteoblasts, endothelial cells, neurological cells)

Using CellPhoneDB2, a cell ligand/receptor pairing-based database,^19^ we identified a close interaction between the monocytes/macrophages and MSCs (Fig. 2a). To further identify the regulatory effects of macrophages on stromal cells, we plotted the essential cytokines that involved in macrophage-MSC crosstalk, including *Tgfb*,^20^ *Osm*,^21^ *Lrp1*^18^*,* *Igf1*^22^, and *Bmps*^23^ (Fig. 2b). We found that *Osm*/*Osmr* and *Osm*/*Lifr* pathways were the most significantly enriched in monocyte/macrophage-MSC crosstalk. *Tgfb* and *Lrp1* related pathways also have a strong interaction with MSCs. However, the expression of *Bmp2*, *Bmp4*, and *Igf1* in alveolar bone monocyte/macrophages was barely detectable (Fig. 2c), indicating a limited role of these pathways in regulating monocyte/macrophage-MSC crosstalk. The expression of corresponding receptors in different types of stromal cells was also shown. *Bmpr1a* and *Mdk* were mainly expressed in MSCs, while other receptors were widely expressed in various cell types (Fig. 2d).

### Comparative analysis of the heterogeneity of monocytes/macrophages

**Fig. 3:**
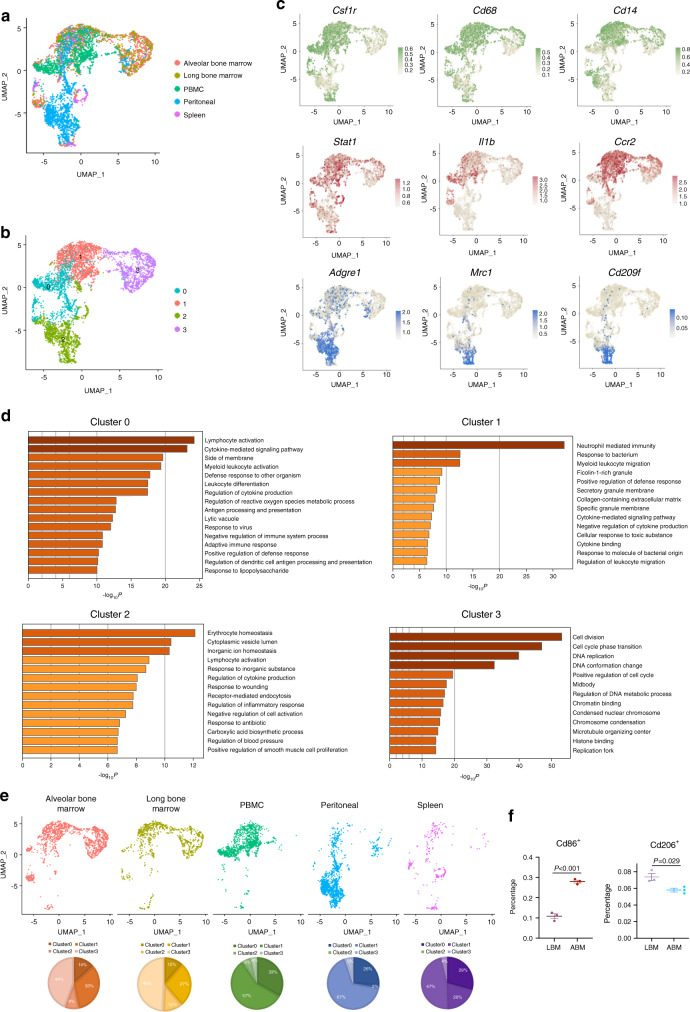
Comparative analysis of the heterogeneity of monocytes/macrophages. **a** Monocyte/macrophage population after merging five scRNA-seq datasets was visualized with umap plot. **b** Identification of the 4 subclusters of Monocyte/Macrophage population. **c** The expression of classic macrophage polarization markers in monocyte/macrophage population. **d** GO enrichment analysis of the biological functions of different subclusters. **e** Distribution of cells on umap plot split by tissue origins. **f** Flow cytometry analysis of the ratio of Cd86^+^ and Cd206^+^ macrophages in alveolar bone marrow and long bone marrow

To unveil the unique characteristics of alveolar bone-derived monocytes/macrophages, we retrieved published scRNA-seq datasets of immune organs (bone marrow, peripheral blood mononuclear cells, peritoneum, and spleen) for comparative analysis (Fig. 3a, Fig. S3a). The *Csf1r*-positive cell population was extracted and identified as monocyte/macrophage (Fig. S3b). The monocyte/macrophage population was divided into four subclusters (Fig. 3b). Clusters 0 and 1 expressed universal markers of monocytes/macrophages, such as *Csf1r*, *Cd68*, and *Cd14* (Fig. 3c). For cluster 1, the polarized macrophage markers, such as *Stat1*, *Il1b*, and *Ccr2*, were highly expressed (Fig. 3c). For cluster 2, markers of alternatively activated macrophages, such as *Mrc1* and *Cd209f*, were highly expressed.

Next, we performed GO enrichment analysis and found that cluster 0 was enriched in phagocytic functions and antigen presentation (Fig. 3d). Cluster 1 was related to cytokine secretion, bacterial and intracellular pathogen immune response. Characteristic features of alternatively polarized macrophages such as wound healing and regulation of blood vessel formation, were found in cluster 2. The biological functions of the cluster 3 were enriched in cell division, DNA replication, and cell proliferation-related pathways. In brief, cluster 3 was the main proliferative population of monocyte/macrophage, and clusters 0, 1, and 2 were mature monocyte/macrophage populations with different functions.

As monocytes/macrophages are highly plastic that can be polarized to different states depending on the tissue microenvironment,^24,25^ we compared the cell composition among monocytes/macrophages from different origins (Fig. 3e). ABM and LBM had the closest cell composition. Cluster 1 accounted for a high proportion of ABM, LBM, and peripheral blood mononuclear cells (PBMC). Interestingly, almost all the cells in PBMC were distributed in cluster 1 and most cells in peritoneum were enriched in cluster 2. Monocytes/macrophages in the spleen were evenly distributed in clusters 0, 1, and 2. Cluster 3 was almost exclusively distributed in bone marrow tissue.

Through flow cytometry analyses of ABM and LBM, we verified that the proportion of Cd86^+^ (a classical polarization marker) macrophages in ABM (~28%) was about twice that of LBM (~11%), while that of Cd206^+^ (alternatively polarization marker) macrophages in ABM was slightly lower (Fig. 3f, Fig. S4). Moreover, through the combined analysis of single-cell sequencing of immune cells in ABM and LBM, we found that the ratio of mature B cells and neutrophils is higher in ABM, and the proportion of pro-B cells and myeloid prog cells is higher in LBM (Fig. S5).

**Fig. 4:**
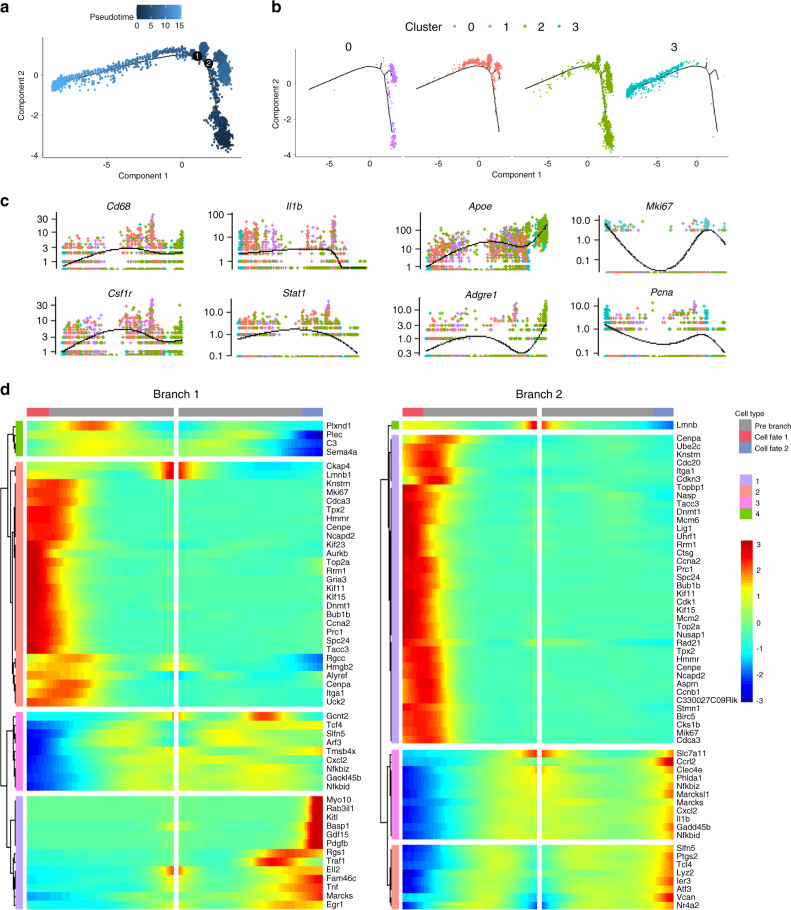
Pseudotime analysis of monocyte/macrophage population. **a** Trajectory order of the monocyte/macrophage populations by pseudotime value. **b** Distribution of monocytes/macrophages on the developmental tree by clusters. **c** Cluster-defined monocyte/macrophage marker gene expression of different subclusters. **d** Clustered heatmap of differential genes at two trajectory branch points (*P* < 1.0−8e)

Based on the gene expression dynamics of monocytes/macrophages, we constructed a pseudotime developmental tree and determined two independent branch points (Fig. 4a). The 4 monocyte/macrophage subclusters scattered at different branches in the developmental tree (Fig. 4b). Cluster 3 had the lowest pseudotime value and was located at the initial position of the developmental tree, indicating a developmental origin for other subclusters. This observation was consistent with the GO enrichment of cluster 3 (as a proliferative population) (Fig. 3d). Cluster 0 and 1 located at two different branches, while cluster 2 distributed more extensively, partially overlapping with clusters 0 and 1.

The gene expression pattern across pseudotime showed that the expression curves of *Cd68* and *Csf1r* were relatively smooth (Fig. 4c). The increase in the expression of *Apoe* and *Adgre1* was accompanied by a decrease in the expression of *Il1b* and *Stat1*, but the expression of *Mki67* and *Pcna* was only upregulated in cluster 3. As shown in Fig. 4d, genes with the most significant changes in the two branches were clustered.

Meanwhile, we found that the expression of *Bmp2* and *Igf1*, which was barely detectable in ABM-derived monocytes/macrophages (Fig. 2c), was significantly increased in other sources of macrophages (in peritoneal macrophages) (Fig. S6a). The expression of several angiogenesis-related growth factors differed within subclusters (Fig. S6b). *Vegfa* and *Pigf* were highly expressed in cluster 1, and *Vcam1* was mainly expressed in cluster 2, suggesting that monocytes/macrophages from different subclusters have different patterns of cytokine secretion.

### Regulatory effect of monocytes/macrophages on MSCs

**Fig. 5:**
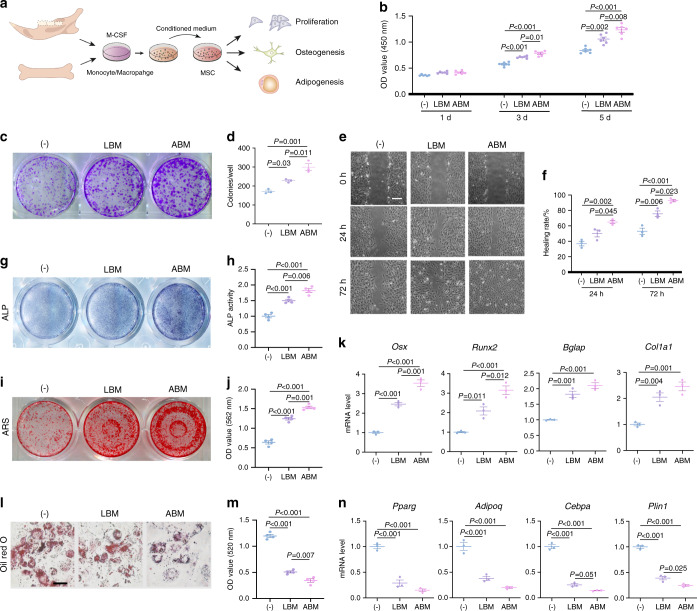
Regulatory effect of monocytes/macrophages on MSCs. **a** Flow chat of the experimental procedures. Conditioned mediums of ABM- and LBM-derived monocytes/macrophages were supplemented into the MSCs culture, respectively. **b** Proliferation assay of MSCs at 1d, 3d, and 5d by CCK8. **c**, **d** Colony formation of MSCs at 14 days via crystal violet staining and quantitative analyses. **e**, **f** Effects of macrophage-conditioned medium on the migration of MSCs. Scale bar, 200 μm. **g**, **h** ALP staining and ALP activity measurement of MSCs after induction for 7 days. **i**, **j** ARS staining and quantitative analyses after 21 days induction. **k** RT-qPCR results for the osteogenesis-related genes after 7 days induction. **l**, **m** Oil red O staining and quantitative analyses of MSCs. Scale bar, 50 μm. **n** RT-qPCR results for the adipogenesis-related genes after 21 days induction

To compare the regulatory effects of ABM- and LBM-derived monocytes/macrophages on MSCs, we supplemented these two types of conditioned mediums into the MSCs culture, respectively (Fig. 5a). Both conditioned mediums promoted the proliferation of MSCs, and ABM-derived one induced a greater proliferation on day 5 (Fig. 5b). Although both conditioned mediums enhanced the colony-forming ability of MSCs, the effect of ABM was more significant (Fig. 5c, d). In addition, scratch assay showed that ABM-derived monocytes/macrophages conditioned medium more efficiently accelerated the migration of MSCs (Fig. 5e, f).

Next, we sought to compare their effect on differentiation of MSCs. Alkaline phosphatase (ALP) staining and Alizarin red (ARS) staining proved that both conditioned mediums promoted the osteogenic differentiation of MSCs (Fig. 5g–j). In addition, the expressions of osteogenesis-related genes (*Osx*, *Runx2*, *Bglap, Col1a1*) were significantly upregulated (Fig. 5k). As for adipogenic differentiation of MSCs, both macrophage-conditioned medium inhibited lipid droplet formation (Fig. 5l, m) and downregulated the expression of adipogenic-related genes (*Pparg*, *Cebpa*, *Adipoq*, *Plin1*) (Fig. 5n). Notably, the conditioned medium from ABM-derived monocytes/macrophages exhibited an enhanced effect in promoting osteogenic differentiation and inhibiting adipogenic differentiation of MSCs, which indicated a different immune-modulatory potential between alveolar bone and long bone.

### Osm is highly expressed in ABM-derived monocytes/macrophages

**Fig. 6:**
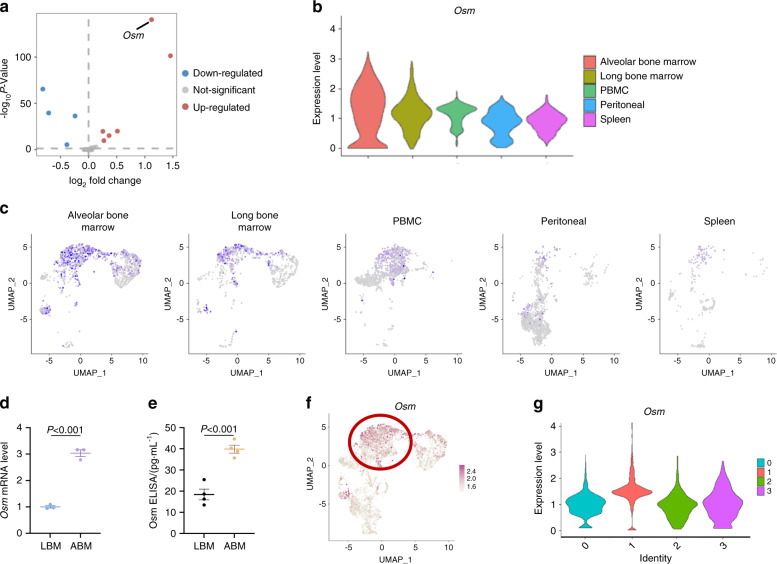
Osm is highly expressed in ABM. **a** The volcano plot shows 59 cytokines between ABM and LBM-derived monocyte/macrophage populations. Red symbol, significantly upregulated cytokines. Blue symbol, significantly downregulated cytokines. **b** Violin plot for the expression levels of *Osm* in monocytes/macrophages from different tissues. **c** *Osm* expression in monocytes/macrophages split by tissue origin. **d** RT-qPCR results of *Osm* levels in monocytes/macrophages derived from ABM and LBM. **e** ELISA for Osm in ABM and LBM homogenates. **f** *Osm* expression levels in different cells projected on UMAP plot. **g** Violin plot for the expression levels of *Osm* in different monocyte/macrophage subclusters

Next, we explored the underlying mechanism by comparing the expression of previously reported 59 cytokines.^26^ *Osm* was identified as the most differently expressed cytokine between ABM and LBM monocyte/macrophage (Fig. 6a). We found that the expression of *Osm* in ABM-derived macrophage was significantly higher than other tissues (Fig. 6b, c). This observation was further confirmed by qRT-PCR analysis and enzyme-linked immunosorbent assay (ELISA) (Fig. 6d, e).

Osm is a regulatory cytokine considered to be secreted by the classically polarized macrophages.^27,28^ Among the four monocyte/macrophage subclusters, we found that *Osm* was mainly expressed in cluster 1 (Fig. 6f, g). Therefore, we supposed that the higher proportion of activated macrophages in ABM led to the increased expression of *Osm*, which subsequently regulate the osteogenic and adipogenic differentiation of MSCs.

### The regulatory effect of monocytes/macrophages on MSCs is Osm-dependent

**Fig. 7:**
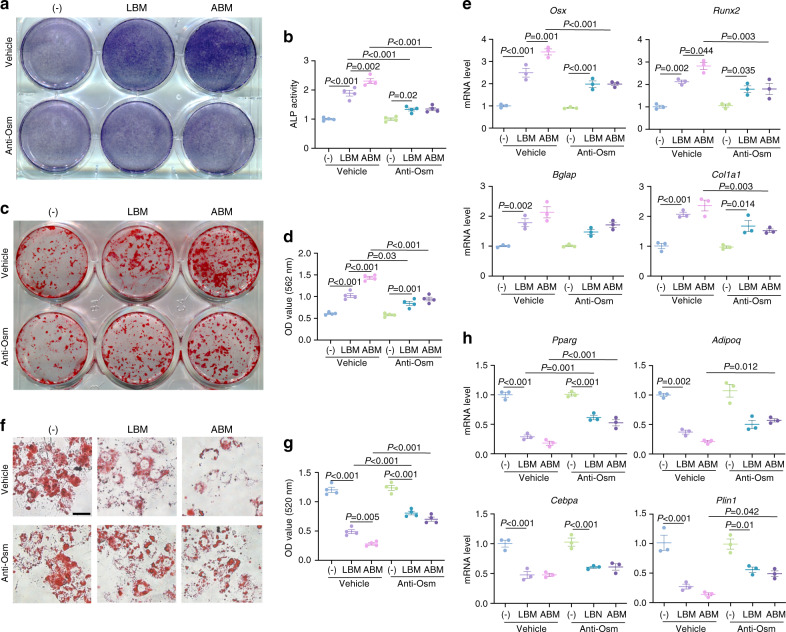
The regulatory effect of macrophages on MSCs is Osm-dependent. **a**, **b** ALP staining and ALP activity quantitative analyses of MSCs after Osm neutralization. **c**, **d** ARS staining and quantitative analyses of MSCs after Osm neutralization. **e** RT-qPCR for the osteogenesis-related genes after Osm neutralization. **f**, **g** Oil red O staining and quantitative analyses of MSCs after Osm neutralization. Scale bar, 50 μm. **h** RT-qPCR for the adipogenesis-related genes after Osm neutralization

To test whether the effect of monocytes/macrophages conditioned medium on the osteogenic differentiation of MSCs was Osm-dependent, we added Osm neutralizing antibody to the conditioned medium. Osm antibody significantly weakened the ALP activity and ARS staining induced by macrophage-conditioned medium (Fig. 7a–d), and the expression of osteogenesis-related genes was also downregulated (Fig. 7e). Osm neutralization partially rescued the lipid droplet formation of MSCs under macrophage-conditioned medium (Fig. 7f, g), and increased the expression of adipogenesis-related genes (Fig. 7h). Notably, after Osm neutralization, ABM and LBM macrophage-conditioned medium showed similar osteogenic and adipogenic potential, suggesting the higher expression of *Osm* in ABM-derived monocytes/macrophages was a key factor that led to the different effects between the ABM and LBM.

## Discussion

The oral environment is resided with a complex microbial community, and periodontal microorganism can penetrate the periodontal barrier to alveolar bone.^29^ Therefore, the immune homeostasis of alveolar bone might be directly affected by microorganisms. Besides, alveolar bone is bearing mechanical loads, whose force is two times higher than that of long bones.^30,31^ A recent study showed that mechanical stimulation could promote the conversion of myeloid-derived monocytes into an activated status,^32^ suggesting that occlusal force might lead to the difference of the immune microenvironment between alveolar bone and long bone.

In this study, we constructed a single-cell atlas of the mouse mandibular alveolar bone by 10x scRNA-seq, and found that immune cells in the mouse mandibular microenvironment accounted for the majority of cell components. We conducted a cell-to-cell communication analysis and found that among various types of immune cells, monocyte/macrophage population had the most interactions with MSC population. Based on previous studies about the regulation of osteogenic differentiation by monocytes and macrophages,^18,22,23,33,34^ we analyzed several previously reported classic pathways for monocytes/macrophages to regulate MSCs, and found *Tgfb*, *Osm*, and *Lrp1* were widely expressed in alveolar bone monocytes/macrophages. As for the expression of cytokine receptors in stromal cells, both *Osmr* and *Lifr* were expressed in MSCs and osteoblasts, while *Osmr* was also highly expressed in endothelial cells. Study has shown that Osm could also promote angiogenesis in vitro and in vivo.^35^ In addition, we found that in the monocyte/macrophage population, the expression of growth factors related to angiogenesis including *Vegfa* and *Pigf* was also higher. Therefore, we suppose that macrophages participate in the maintenance of bone homeostasis by regulating both osteogenesis and angiogenesis.

The monocyte/macrophages were divided into four main subclusters, and the proportions of each subcluster from different tissues were quite different. Compared with long bone, the proportion of the cluster 1 (classically polarized monocyte/macrophage) in the ABM was relatively higher. Interestingly, the proportion of cluster 2 (alternatively polarized monocyte/macrophage) was very low in both ABM and LBM. Monocytes/macrophages in bone marrow and peripheral blood are mainly derived from bone marrow HSCs, while monocytes/macrophages in peritoneum are generally considered as tissue-resident macrophages. The different tissue sources and microenvironment have significant effects on the state of monocyte/macrophages.^33,36^ In this study, we found that the monocyte/macrophage population in bone marrow microenvironment participates in the regulation of MSCs and bone homeostasis by expressing *Tgfb*, *Osm*, and *Lrp1* rather than *Bmps* and *Wnts*.

Previous study revealed that monocyte/macrophage-conditioned medium could promote MSCs colony formation in vitro.^18^ In this study, we observed that ABM-derived monocyte/macrophage-conditioned medium more effectively promote the proliferation and migration of MSCs, meanwhile enhancing osteogenic differentiation and inhibiting adipogenic differentiation. Although the monocyte/macrophage population may lose part of the cell heterogeneity during in vitro culture, it can still reflect the different regulatory effects of monocyte/macrophage derived from alveolar bone compared to long bone. To explore the mechanism responsible for their different regulatory effects, we compared the expression of cytokines in ABM and LBM monocytes/macrophages through differential gene analysis. We found *Osm* is most significantly upregulated among the 59 cytokines.

Osm is a cytokine secreted by monocytes/macrophages, and has important regulatory role on bone homeostasis.^37–40^ *Osm* receptor knockout mice exhibit decreased bone remodeling activity.^34^ In a tibial defect model, *Osm* knockout led to a decrease in osteoblasts number and delayed bone healing.^41^ Moreover, Osm inhibited the adipogenic differentiation of MSCs.^42^ *Osm* knockout mice show increased adipose tissue accumulation in bone marrow with age.^43^ Therefore, we speculate that the higher expression of Osm secreted by monocyte/macrophage in ABM may regulate the fate commitment of MSCs, leading to different biological characteristics of alveolar bone and long bone. In addition, Osm has an indirect role on osteoclasts, because it is one of the most important cytokines that stimulates osteoblasts to secrete receptor activator of nuclear factor-kappa B ligand (RANKL).^44^ We, therefore, speculate that the high expression of Osm might induce more active osteoclasts in the alveolar bone to participating alveolar bone remodeling. Further study is expected to elucidate the role of Osm on osteoclast in ABM.

In summary, we constructed a single-cell atlas of mouse mandibular alveolar bone through 10x scRNA-seq, and verified the regulatory effect of monocytes/macrophages on MSCs. Our study reveals a unique immune microenvironment of alveolar bone, which might provide a more precise immune-modulatory target for therapeutic treatment of oral diseases.

## Materials and methods

### Single-cell RNA sequencing

Eight 12-week-old male C57BL/6J wild-type mice (Chengdu Dossy Experimental Animals CO.LTD) were combined to extract single-cell suspensions. Mandible was carefully dissected under a stereo microscope to obtain the mandibular alveolar bone tissue. Soft tissues and molars are removed. Jaw bone around the incisor and behind condyle was cut off. Subsequently, the obtained alveolar bone tissue was cut into small pieces (<1 mm^3^) and digested in 1 mg·mL^−1^ collagenase type I (Worthington) and 1 mg·mL^−1^ Dispase II (Sigma-Aldrich) at 37 °C for 1 h (200 r·min^−1^). The sample was then collected into a centrifuge tube through a 40-μm filter, and the supernatant was removed after centrifugation. 1 mL of ACK lysis buffer was added on ice for 5 min to lyse red blood cells, and the supernatant was removed by centrifugation.

The centrifuged cells were resuspended in 0.4% BSA PBS solution. Placenta blue stain was used to calculate the number of cells and cell viability on a hemocytometer, and the cell concentration was adjusted to 800–1 500 cells per μL. About 20 000 cells were loaded to capture 10 000 cells. cDNA library was constructed using Chromium single-cell v3.0 reagent, and sequenced on the Illumina Nova-seq system.

### Preprocessing of scRNA-seq data

After obtaining the initial sequencing data, we compared them to the mouse genome mm10, and folded the UMI with Cellranger (version 3.1, 10x Genomics) software to obtain a single-cell gene expression matrix. Then we imported the expression matrix into the Seurat package (v 4.0) for further analysis. Genes expressed in less than three cells are deleted, mitochondrial genes >25%, and cells with genes <300 are filtered out. The FindVariableGenes function in the Seurat package is used to select variable genes, and then principal component analysis (PCA) was performed, and UMAP dimensionality reduction and visualization were performed based on PCA results. According to the specific genes of different subgroups, we annotated the cell types of different subclusters.

### Cell–cell interaction analysis

CellPhoneDB2 is a Python-based analysis tool for calculating the interaction between ligands and receptors between different cell populations. So far, CellPhoneDB2 only supports the input of human genes. Therefore, we first mapped the mouse mgi_symbol to human hgnc_symbol through the BioMart package, and then used CellPhoneDB2 for cell–cell communication calculation and analysis. To reveal the strength of the specific pathways between macrophages and MSCs, we selected several common macrophage-MSC-related pathways for visualization, including *Bmps*, *Wnts*, *Osm*, *Tgfb*, and *Lrp1*.

### Pseudotime analysis

To determine the differentiation trajectory of monocytes/macrophages, we used Monocle2 package to calculate the differentiation trajectory of these cells. After determining the pseudotime value arrangement and differentiation trajectory, we used the plot_genes_in_pseudotime function in the Monocle2 package to show the changes of classic macrophage marker genes in the differentiation trajectory. The clustered heatmap of the expression pattern of the hub genes (*P* < 1e–8) in branches 1 and 2 was displayed with the plot_pseudotime heatmap function.

### Flow cytometry analysis

The mouse mandibular alveolar bone and femur were obtained according to the above manner. Then the alveolar bone and femur were cut into pieces, digested in 1 mg·mL^−1^ collagenase I, 1 mg·mL^−1^ dispase II at 37 °C for 30 min, centrifuged. After lysis of red blood cells on ice, the samples were passed through a 70-μm filter, centrifuged, and ready for staining. FITC anti-mouse Cd11b, PE-Cy7 anti-mouse Cd86, and APC anti-mouse Cd206 were purchased from BD Biosciences, and the permeabilization/fixation kit was purchased from eBioscience. All staining processes were performed in 100 μL PBS. For cell surface staining, after blocking the cell surface Fc receptors, the flow cytometry antibody was directly added to the cell suspension and stained on ice for 30 min. For intracellular antibody staining, the fixed cells were permeabilized and then stained with flow cytometry antibody for 30 min. The samples were then tested using flow cytometry (BD Biosciences), and the flow cytometry data were analyzed and visualized using Flowjo software.

### Culture of bone marrow MSCs

To isolate MSCs, we separated the femur and tibia from C57BL/6J mice in PBS, the bone marrow was flushed out and transferred to α-MEM medium containing 10% FBS (Gibco) and 1% penicillin/streptomycin (Hyclone). After culturing in a petri dish with a diameter of 100 mm at 37 °C, a 5% CO_2_ constant temperature incubator for 48 h, the medium was changed to remove non-adherent cells.^45^ Afterward, the medium was changed every 3 days and digested when the confluence reached 80% for the next experiment.

### Isolation of monocytes/macrophages

To isolate macrophages from long bone, we dissected the femur and tibia as described above and flush out the bone marrow to mix with the culture medium. For macrophages from ABM, we separated the alveolar bone as described above and then cut the alveolar bone into small pieces in the culture medium. After the bone pieces settled, the culture medium containing the bone marrow cells was transferred. To balance the number of primary cells extracted from mouse alveolar bone and femur, the mandibular alveolar bones of 10 mice were combined. Then the isolated cells were cultured with 10% FBS and 1% penicillin/streptomycin αMEM medium, and 50 ng·mL^−1^ M-CSF (R&D Systems) was added.^46^ The medium was changed three days later to remove non-adherent cells.

To obtain the monocyte/macrophage-conditioned medium, the medium was changed once every three days and the conditioned medium was collected, centrifuged at 1 500 × *g* for 10 min. The supernatant was stored for further use. For conditioned medium treatment, the conditioned medium collected from the ABM and LBM monocyte/macrophage directly mixed with the complete culture medium, osteogenic induction medium, and adipogenic induction medium at a ratio of 1:1. For wound healing analysis, 1% FBS αMEM medium was used to eliminate the influence of serum on cell migration.

### Cell proliferation assay

The MSCs were seeded in a 96-well plate at a density of 5 000 cells per well, and the conditioned medium for alveolar bone and long bone was added as described above. After culturing for 1 d, 3 d, and 5 d, 10 μL of CCK8 (Dojindo Laboratories) reagent was added to each well and then placed in the incubator to continue incubating for 1 h, and the absorbance at 450 nm was measured with a microplate reader.

### Colony formation test

MSCs were seeded in 6-well plate at a density of 500 cells per well. ABM and LBM-derived macrophage-conditioned medium was added as described above, and the medium was changed every 3 days. After 2 weeks of culture, cells were fixed with 4% paraformaldehyde solution and stained with 1% crystal violet solution. Colonies with more than 30 cells were included.

### Scratch wound healing assay

MSCs were seeded in 6-well plate and cultured till 100% confluence, and the 10% FBS medium was replaced with 1% FBS medium for starvation culture 24 h before making scratches. A 20–200 μL pipette tip was applied to trace the center of the orifice along a ruler, leaving a uniform and straight scratch.^47^ Subsequently, the medium was replaced with 1% FBS medium and macrophage-conditioned medium. The wound closure was observed and pictures were taken at 0/24/72 h. ImageJ software was used to calculate the proportion of the healing area.

### Osteogenic and adipogenic differentiation

MSCs derived from long bone marrow (femur and tibia) were used for all subsequent studies. For osteogenic induction, MSCs were seeded into a 24-well plate at a density of 2.5 × 10^5^ per well. Ascorbic acid (50 μg·mL^−1^), β-glycerophosphate (5 mmol·L^−1^), and dexamethasone (100 nmol·L^−1^) were added to αMEM complete medium.^48^ Conditioned medium was added at a ratio of 1:1.

After 7 days of induction, ALP staining and ALP activity detection were performed. ALP staining was performed following the recommended protocol with ALP staining kit (Beyotime). After incubating for 15 min, a scanner (EPSON) was used to collect images. ALP activity was measured by ALP assay kit (Nanjing Jiancheng) according to the recommended protocol.^49^

ARS staining was performed 21 days after induction. 1% Alizarin Red Staining Solution (Solarbio) was used to stain the mineralized nodules. After fixing the cells for 15 min, the excess staining solution was washed off with PBS, and the stained image was obtained by scanning under the scanner. Subsequently, 10% cetylpyridinium chloride was used to dissolve the mineralization, and the absorbance was detected at 562 nm with a microplate reader (Thermo Fisher).^50^

For adipogenesis induction, MSCs were seeded into 24-well plates at a density of 2.5 × 10^5^ per well. 3-isobutyl-1-methylxanthine (IBMX, 0.5 mmol·L^−1^), dexamethasone (1 μmol·L^−1^), and insulin (10 μg·mL^−1^) were added to the DMEM complete medium.^51^ The conditioned medium was added at a ratio of 1:1. After 21 days of induction, the cells were fixed with 4% paraformaldehyde solution, and then the intracellular lipid droplets were stained with 1% oil red O solution. The excess dye solution was washed away with PBS, and the images were taken with an inverted microscope. Then isopropanol was used to dissolve the Oil Red O dye, and the absorbance was measured at a wavelength of 520 nm using a microplate reader.

### qPCR

The total RNA was isolated with Trizol reagent (Invitrogen), and the absorbance A260/A280 was measured with Nanodrop 2000 to detect the concentration and purity. PrimeScript RT kit (TaKaRa Bio) was used to reverse transcription of RNA into cDNA and added to a PCR instrument (Bio-Rad) for real-time RT-PCR. Gapdh was used as an endogenous control, and the relative expression level of mRNA was calculated by the 2^−ΔΔCt^ method. For adipogenesis-related genes, 34B4 was used as an endogenous control.

### ELISA

Osm levels in alveolar bone and long bone tissue were measured by a mouse Osm ELISA kit (Cusabio). Briefly, mouse alveolar bone and long bone were collected, cut into pieces, and weighed for the same weight (100 mg). the samples were diluted into 1 mL with buffer, frozen, and thawed three times in a −80 °C refrigerator. After centrifugation at 200 × *g* for 5 min, 100 μL of supernatant was added to a 96-well plate with high binding capacity and incubated for 2 h.

### Statistical analysis

All values were expressed as mean ± SEM. Statistically significant differences were performed by two-tailed Student’s *t* test for comparison between two groups, one-way or two-way analysis of variance (ANOVA) followed by the Tukey’s post hoc test for multiple comparisons. *P* value < 0.05 was considered to be statistically significant.

## Supplementary information

Supplementary figure 1-6

## Data Availability

Mouse alveolar bone scRNA-seq data could be downloaded from Sequence Read Archive (SRA) datasets (https://www.ncbi.nlm.nih.gov/sra/) via accession number PRJNA697839. All public scRNA-seq dataset used in the study, including long bone marrow cells (GSE109774), peritoneal macrophages (GSE139999), peripheral blood mononuclear cells (GSE108097), and spleen cells (GSE109774), can all be downloaded from the GEO database (https://www.ncbi.nlm.nih.gov/geo/).

## References

[1] Gruber R (2019). Osteoimmunology: Inflammatory osteolysis and regeneration of the alveolar bone. J. Clin. Periodontol..

[2] Lerner UH, Kindstedt E, Lundberg P (2019). The critical interplay between bone resorbing and bone forming cells. J. Clin. Periodontol..

[3] Connizzo BK (2021). Nonuniformity in periodontal ligament: mechanics and matrix composition. J. Dental Res..

[4] Huja SS, Fernandez SA, Hill KJ, Li Y (2006). Remodeling dynamics in the alveolar process in skeletally mature dogs. Anat. Rec. Part A, Discoveries Mol. Cell.Evolut. Biol..

[5] Chai Y (2000). Fate of the mammalian cranial neural crest during tooth and mandibular morphogenesis. Development.

[6] Matsubara T (2005). Alveolar bone marrow as a cell source for regenerative medicine: differences between alveolar and iliac bone marrow stromal cells. J. Bone Miner. Res..

[7] Aghaloo TL (2010). Osteogenic potential of mandibular vs. long-bone marrow stromal cells. J. Dent. Res..

[8] Mavropoulos A, Rizzoli R, Ammann P (2007). Different responsiveness of alveolar and tibial bone to bone loss stimuli. J. Bone Miner. Res..

[9] Coutel X (2019). Mandibular bone is protected against microarchitectural alterations and bone marrow adipose conversion in ovariectomized rats. Bone.

[10] Tsukasaki M (2021). RANKL and osteoimmunology in periodontitis. J. Bone Miner. Metab..

[11] Baryawno N (2019). A cellular taxonomy of the bone marrow stroma in homeostasis and leukemia. Cell.

[12] Tikhonova AN (2019). The bone marrow microenvironment at single-cell resolution. Nature.

[13] Baccin C (2020). Combined single-cell and spatial transcriptomics reveal the molecular, cellular and spatial bone marrow niche organization. Nat. Cell Biol..

[14] Tsukasaki M, Takayanagi H (2019). Osteoimmunology: evolving concepts in bone-immune interactions in health and disease. Nat. Rev. Immunol..

[15] Chang MK (2008). Osteal tissue macrophages are intercalated throughout human and mouse bone lining tissues and regulate osteoblast function in vitro and in vivo. J. Immunol..

[16] Liu A (2020). Macrophage-derived small extracellular vesicles promote biomimetic mineralized collagen-mediated endogenous bone regeneration. Int. J. Oral. Sci..

[17] Vi L (2015). Macrophages promote osteoblastic differentiation in-vivo: implications in fracture repair and bone homeostasis. J. Bone Miner. Res..

[18] Vi L (2018). Macrophage cells secrete factors including LRP1 that orchestrate the rejuvenation of bone repair in mice. Nat. Commun..

[19] Efremova M, Vento-Tormo M, Teichmann SA, Vento-Tormo R (2020). CellPhoneDB: inferring cell-cell communication from combined expression of multi-subunit ligand-receptor complexes. Nat. Protoc..

[20] Sorkin M (2020). Regulation of heterotopic ossification by monocytes in a mouse model of aberrant wound healing. Nat. Commun..

[21] Walker EC (2010). Oncostatin M promotes bone formation independently of resorption when signaling through leukemia inhibitory factor receptor in mice. J. Clin. Investig..

[22] Gong L, Zhao Y, Zhang Y, Ruan Z (2016). The macrophage polarization regulates MSC osteoblast differentiation in vitro. Ann. Clin. Lab. Sci..

[23] Talati M (2014). BMP pathway regulation of and by macrophages. PLoS ONE.

[24] Lawrence T, Natoli G (2011). Transcriptional regulation of macrophage polarization: enabling diversity with identity. Nat. Rev. Immunol..

[25] Russell DG, Huang L, VanderVen BC (2019). Immunometabolism at the interface between macrophages and pathogens. Nat. Rev. Immunol..

[26] Santoso CS (2020). Comprehensive mapping of the human cytokine gene regulatory network. Nucleic Acids Res..

[27] Mommert S, Hüer M, Schaper-Gerhardt K, Gutzmer R, Werfel T (2020). Histamine up-regulates oncostatin M expression in human M1 macrophages. Br. J. Pharmacol..

[28] Tedesco S (2020). Pharmacologic PPAR-γ activation reprograms bone marrow macrophages and partially rescues HSPC mobilization in human and murine diabetes. Diabetes.

[29] Lamont RJ, Koo H, Hajishengallis G (2018). The oral microbiota: dynamic communities and host interactions. Nat. Rev. Microbiol..

[30] Ehrlich PJ, Lanyon LE (2002). Mechanical strain and bone cell function: a review. Osteoporos. Int..

[31] Daegling DJ, Hylander WL (1997). Occlusal forces and mandibular bone strain: is the primate jaw “overdesigned”?. J. Hum. Evol..

[32] Solis AG (2019). Mechanosensation of cyclical force by PIEZO1 is essential for innate immunity. Nature.

[33] Ginhoux F, Jung S (2014). Monocytes and macrophages: developmental pathways and tissue homeostasis. Nat. Rev. Immunol..

[34] Walker EC (2016). Murine oncostatin M acts via leukemia inhibitory factor receptor to phosphorylate signal transducer and activator of transcription 3 (STAT3) but not STAT1, an effect that protects bone mass. J. Biol. Chem..

[35] Vasse M (1999). Oncostatin M induces angiogenesis in vitro and in vivo. Arteriosclerosis, Thrombosis, Vasc. Biol..

[36] Guilliams M, Scott CL (2017). Does niche competition determine the origin of tissue-resident macrophages?. Nat. Rev. Immunol..

[37] Li Y (2020). gp130 controls cardiomyocyte proliferation and heart regeneration. Circulation.

[38] Sharanek A (2020). OSMR controls glioma stem cell respiration and confers resistance of glioblastoma to ionizing radiation. Nat. Commun..

[39] Tan L (2020). Engineered probiotics biofilm enhances osseointegration via immunoregulation and anti-infection. Sci. Adv..

[40] Tseng HW (2020). Neurogenic heterotopic ossifications develop independently of granulocyte colony-stimulating factor and neutrophils. J. Bone Miner. Res.

[41] Guihard P (2015). Oncostatin m, an inflammatory cytokine produced by macrophages, supports intramembranous bone healing in a mouse model of tibia injury. Am. J. Pathol..

[42] Song HY, Jeon ES, Kim JI, Jung JS, Kim JH (2007). Oncostatin M promotes osteogenesis and suppresses adipogenic differentiation of human adipose tissue-derived mesenchymal stem cells. J. Cell. Biochem..

[43] Sato F, Miyaoka Y, Miyajima A, Tanaka M (2014). Oncostatin M maintains the hematopoietic microenvironment in the bone marrow by modulating adipogenesis and osteogenesis. PLoS ONE.

[44] Persson E (2019). Activation of Shc1 allows oncostatin M to induce RANKL and osteoclast formation more effectively than leukemia inhibitory factor. Front. Immunol..

[45] Wu Y (2018). Mettl3-mediated m(6)A RNA methylation regulates the fate of bone marrow mesenchymal stem cells and osteoporosis. Nat. Commun..

[46] Li Q (2020). Ubiquitin-specific protease 34 inhibits osteoclast differentiation by regulating NF-κB signaling. J. Bone Miner. Res..

[47] Sheng R (2021). METTL3-mediated m(6) A mRNA methylation modulates tooth root formation by affecting NFIC translation. J. Bone Miner Res..

[48] Wang Y (2020). Alpha-ketoglutarate ameliorates age-related osteoporosis via regulating histone methylations. Nat. Commun..

[49] Guo YC (2018). Ubiquitin-specific protease USP34 controls osteogenic differentiation and bone formation by regulating BMP2 signaling. EMBO J..

[50] Liu W (2016). GDF11 decreases bone mass by stimulating osteoclastogenesis and inhibiting osteoblast differentiation. Nat. Commun..

[51] Chen Y (2020). AFF1 inhibits adipogenic differentiation via targeting TGM2 transcription. Cell Prolif..

