# Supplementary material for: Mapping the immune microenvironment for mandibular alveolar bone homeostasis at single-cell resolution
Source: Bone Res. 2021 Mar 15;9:17. doi: 10.1038/s41413-021-00141-5 (PMC7960742; doi:10.1038/s41413-021-00141-5)
Supplement: Supplementary file 1 — Supplementary figure 1-6 [file 41413_2021_141_MOESM1_ESM.pdf]

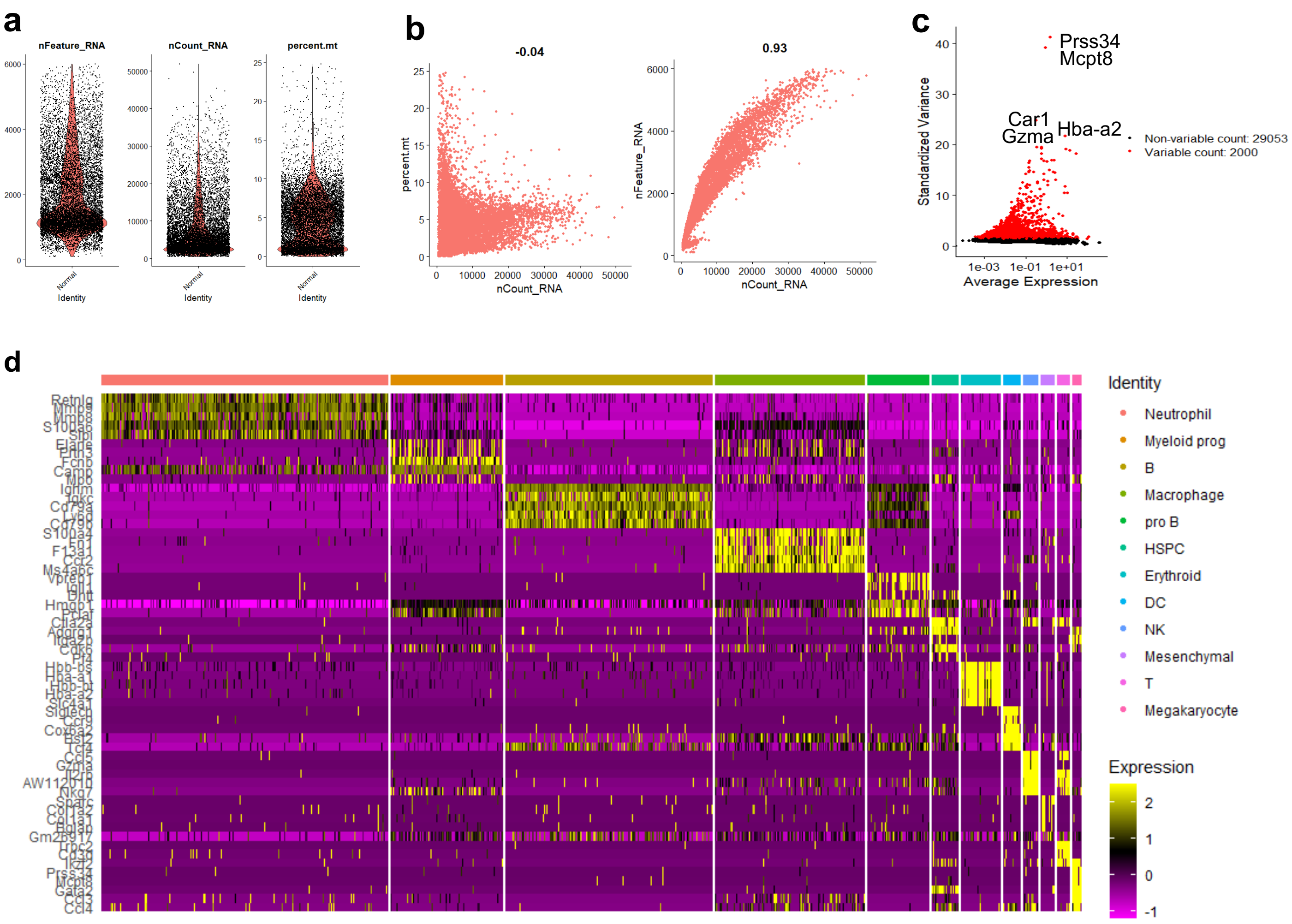

Supplementary figure 1. Quality control of the alveolar bone marrow scRNA-seq data. **a** The violin plots showed the distribution of feature_RNA, count_RNA and mitochondrial RNA proportions. **b** FeatureScatter plot revealed the relationship between mitochondrial RNA and count_RNA, as well as Feature_RNA and count_RNA. **c** The variable feature plot showed the expression levels of 29 053 genes in all cells. The 2000 genes with the most variable value were selected for subsequent analysis, and the top 5 genes were marked. **d** Heatmap of 12 cell subtypes. After dividing the cells into 12 subtypes, top 5 genes with the highest expression in each subtype were identified and compared between the subtypes.

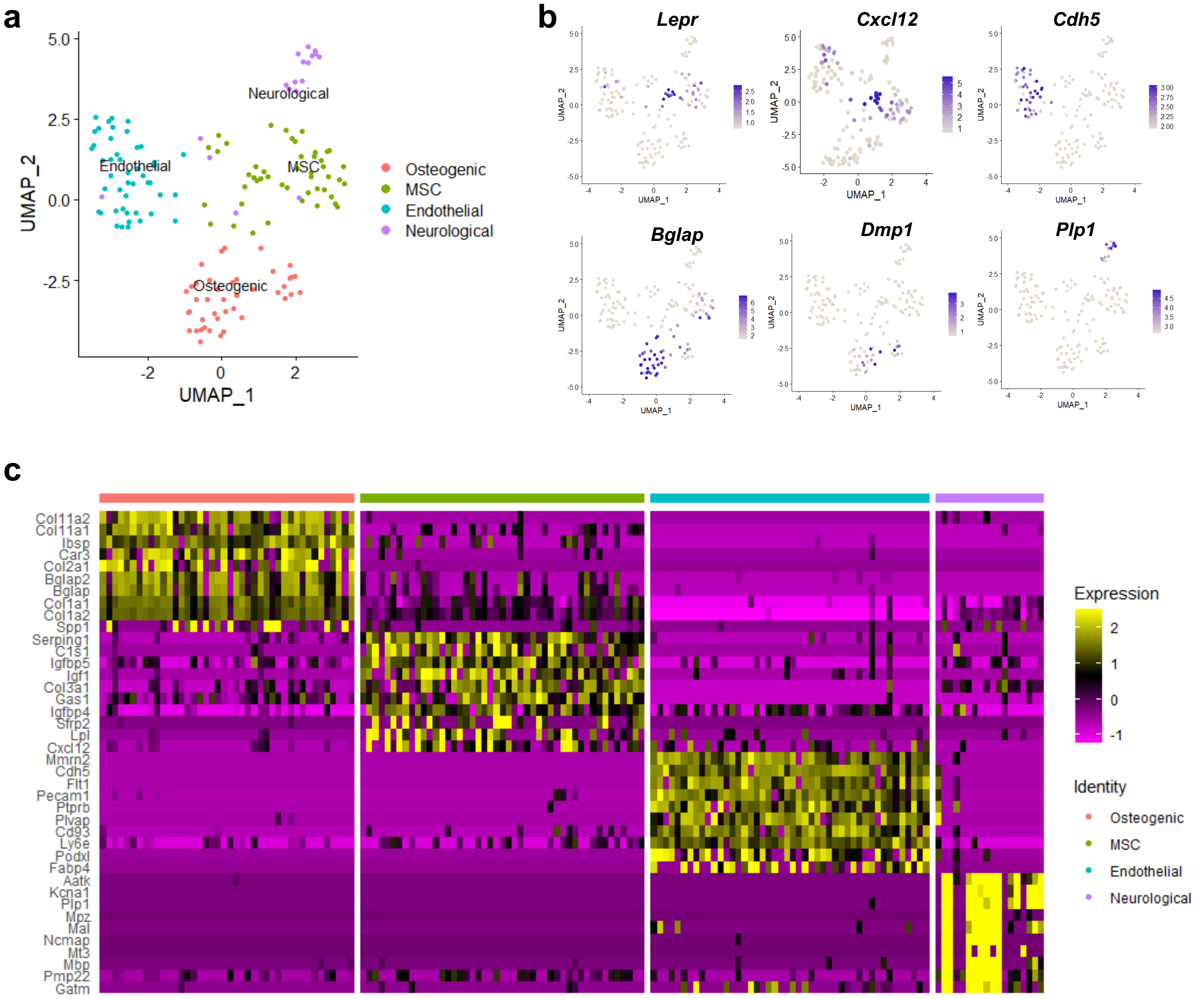

Supplementary figure 2. **Identification of the mesenchymal stromal cell subclusters. a** The expression of marker genes in different stromal cell types were projected onto umap plots. **b** Heatmap of the 4 stromal cell subtypes. Top 5 genes with the highest expression in each subtype were identified and compared between the subtypes.

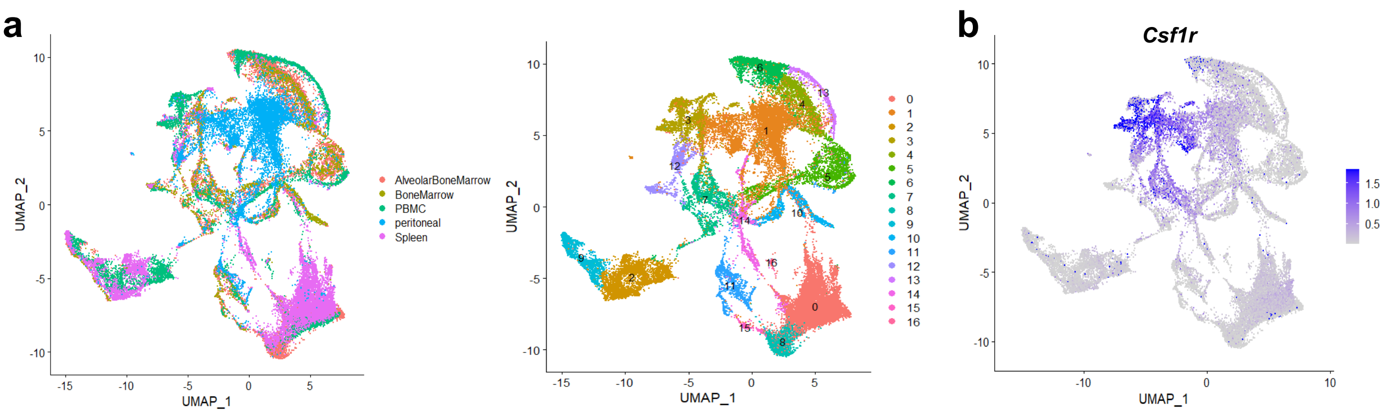

Supplementary figure 3. Combined analyses of scRNA-seq datasets from different origins. a The five scRNA-seq datasets were combined and plotted. **b** Expression of *Csf1r*, a marker gene of monocyte/macrophage population, in the combined five scRNA-seq datasets.

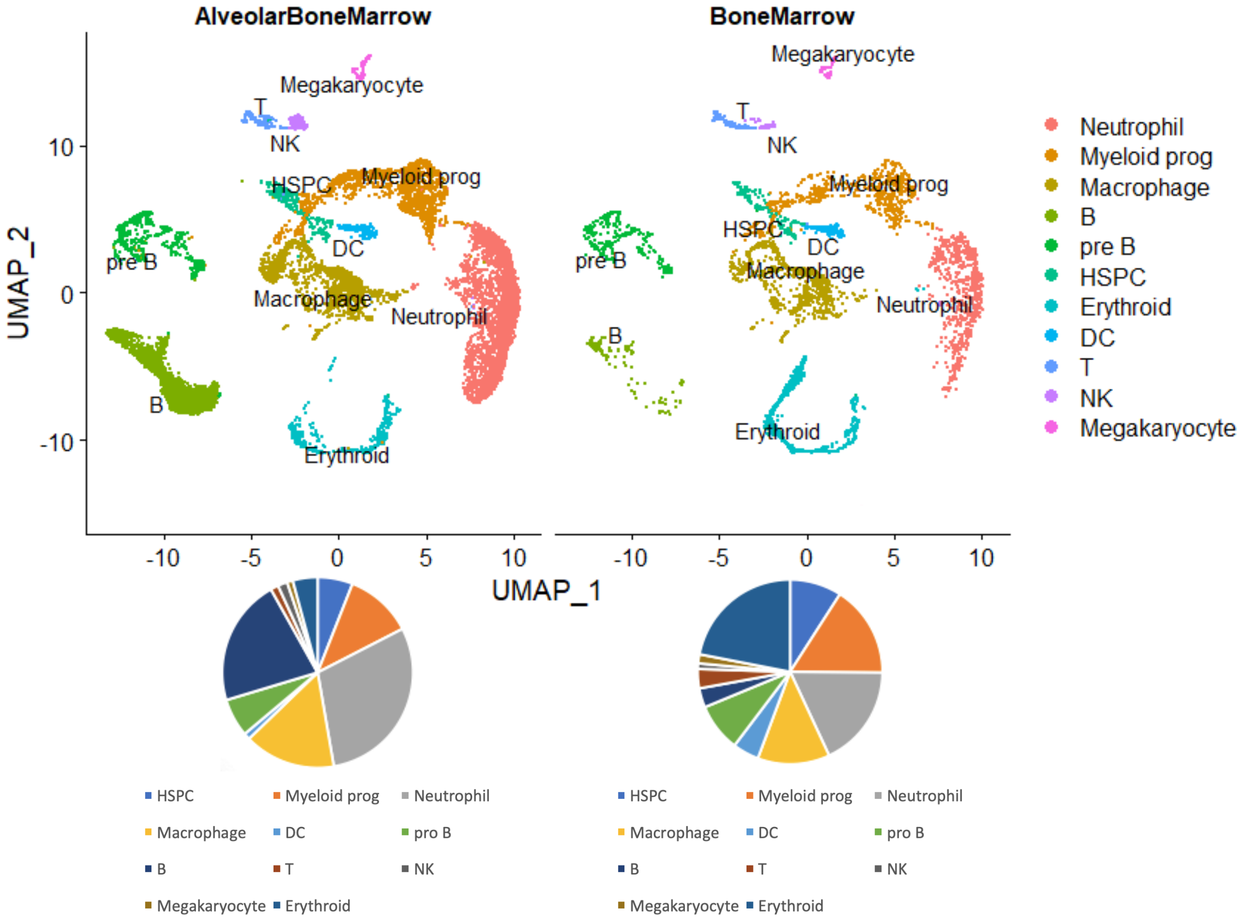

Supplementary figure 4. Comparison of the composition of immune cells between ABM and LBM. The ratios of the 11 immune cell types to the total cell composition were displayed via pie plots.

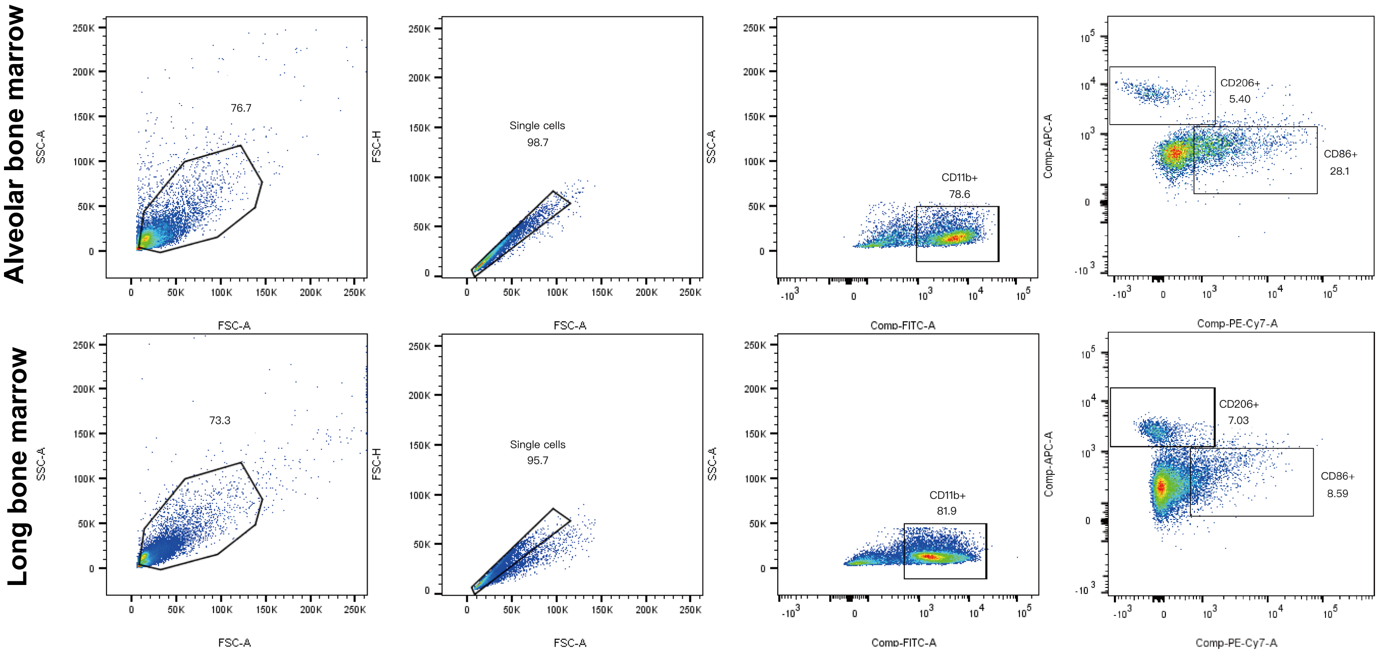

Supplementary figure 5. Flow cytometry analyses of CD86+ and CD206+ macrophages in LBM and ABM.

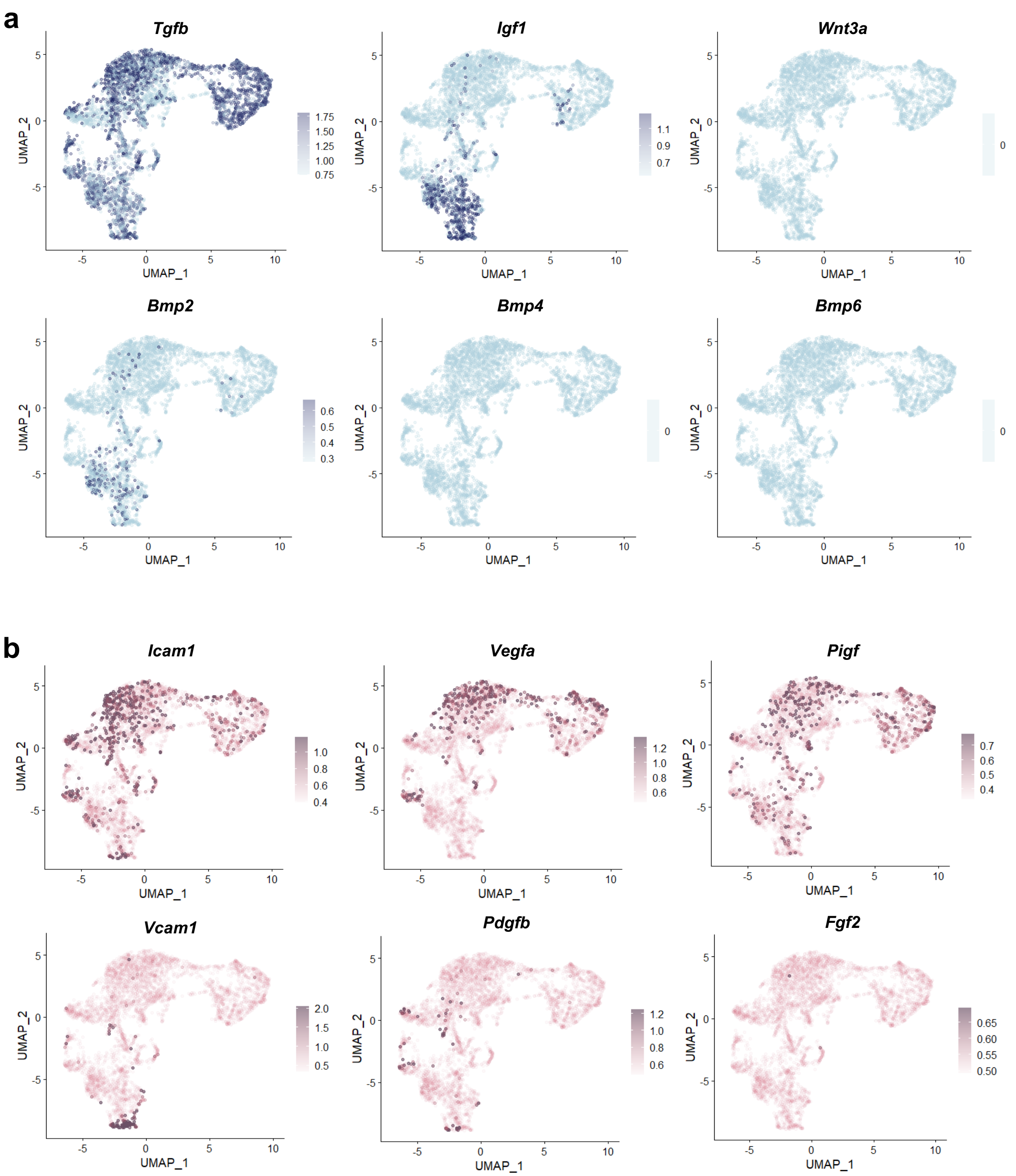

Supplementary figure 6. Expression of cytokines in monocyte/macrophage population. **a** Expression of classic osteogenic-related cytokines. **b** The expression of classic angiogenesis-related cytokines.
